# Ultrasound-detected renal surface nodularity and renal damage in hypertensive adults undergoing routine health screening: a cross-sectional study

**DOI:** 10.3389/fendo.2026.1934293

**Published:** 2026-10-19

**Authors:** Xin Wang, Yue Chen, Hao Zhu, Qingjian Xu

**Affiliations:** 1 Department of Ultrasound, Wuxi Second Hospital of Traditional Chinese Medicine, Wuxi, Jiangsu, China; 2 Department of Ultrasound, Taihu Lake Cadre Sanatorium of Jiangsu Province, Wuxi, China

**Keywords:** arterial hypertension, estimated glomerular filtration rate, nephrosclerosis, renal damage, renal surface nodularity, ultrasound

## Abstract

**Objectives:**

To develop an ultrasound-based grading system for renal surface nodularity (RSN), assess its reproducibility, and evaluate its association with renal damage in hypertensive adults undergoing routine health screening.

**Methods:**

This retrospective cross-sectional study included 871 hypertensive adults who underwent renal ultrasound during routine health screening between October 2025 and March 2026. RSN was graded from stored standardized coronal-plane images on a three-point scale: grade 0 (smooth), grade 1 (mild, undulating), and grade 2 (severe, serrated). Multivariable logistic regression was used to identify factors independently associated with RSN and to evaluate the association between RSN grade and renal damage, defined as eGFR < 60 mL/min/1.73 m² or proteinuria (≥ 1+), with sequential adjustment that included eGFR as a continuous variable in the final model.

**Results:**

RSN was detected in 410 of 871 patients (47.1%): 226 (25.9%) had grade 1 and 184 (21.1%) had grade 2 RSN. Interobserver and intraobserver agreement for image interpretation showed weighted κ values of 0.798 and 0.888, respectively (n = 80). In multivariable analysis, hypertension grade (OR, 2.40; P <.001), hypertension duration (OR, 1.62; P <.001), cortical echo intensity (OR, 1.63; P <.001), and lower eGFR (OR, 0.625 per 10 mL/min/1.73 m² increase; 95% CI: 0.53–0.74; P <.001) were independently associated with RSN (AUC = 0.815). Renal damage was present in 5.2%, 8.0%, and 27.2% of patients with RSN grades 0, 1, and 2, respectively (P for trend <.001). Grade 2 RSN remained associated with renal damage after adjustment for age, sex, hypertension grade, and diabetes (OR, 8.77; 95% CI: 4.49–17.13; P <.001) and after further adjustment for eGFR as a continuous variable (OR, 2.88; 95% CI: 1.26–6.61; P = .012). In a sensitivity analysis restricted to the non-eGFR components of the endpoint and adjusted for continuous eGFR, the association was attenuated and no longer significant (OR, 1.55; P = .30). Among the 779 patients without threshold-defined renal damage, grade 2 RSN was present in 134 (17.2%), and eGFR declined progressively across RSN grades (medians, 98.8, 93.1, and 87.4 mL/min/1.73 m²; P <.001).

**Conclusions:**

Ultrasound can detect and grade RSN, and RSN grade shows a dose–response association with renal damage in hypertensive adults. RSN was frequently present in patients whose eGFR and urinalysis remained within normal limits, suggesting that it may reflect structural change accompanying subclinical decline in renal function.

## Introduction

Hypertensive nephropathy is a leading cause of chronic kidney disease (CKD) and end-stage renal disease worldwide (1, 2). Its pathogenesis involves arteriolar hyalinosis and myointimal hyperplasia, which lead to glomerular ischemia, nephron loss, and interstitial fibrosis (3, 4). Because these changes are largely irreversible, early detection of structural renal change—before clinically apparent dysfunction develops—is essential for timely intervention (5).

Clinical assessment of hypertensive renal damage currently depends on functional biomarkers such as estimated glomerular filtration rate (eGFR) and urinary albumin excretion (6). These indicators have well-recognized limitations: substantial structural damage may already be present before functional biomarkers become abnormal (7, 8). Although kidney biopsy remains the reference standard for diagnosing hypertensive nephropathy, its invasiveness precludes routine clinical use (9).

Imaging offers a noninvasive alternative for detecting structural renal change. CT provides high spatial resolution but requires ionizing radiation and iodinated contrast material. Ultrasound is widely accessible, radiation-free, and cost-effective, making it well suited for clinical screening; however, conventional grayscale ultrasound parameters—renal length, cortical thickness, and cortical echogenicity—have shown limited sensitivity for detecting early CKD-related structural change (10–12).

In this context, renal surface nodularity (RSN) has emerged as a morphology-based imaging phenotype. RSN was first described by investigators at the Mayo Clinic, who demonstrated that microscopic nephron hypertrophy and nephrosclerosis manifest macroscopically as increased renal surface roughness and nodular contour change (13, 14). This nephrosclerotic remodeling is further amplified by mineralocorticoid excess: aldosterone-mediated activation of the mineralocorticoid receptor promotes renal oxidative stress, inflammation, and interstitial fibrosis, and higher cumulative aldosterone exposure has been associated with incident chronic kidney disease, structural renal changes, and markers of renal injury in hypertensive patients (15–17). Subsequent studies reported associations of CT-based RSN with arterial hypertension (18), early renal injury (OR, 14.37 for elevated cystatin C) (19), adverse vascular events (20), decreased eGFR (21), and progression of renal dysfunction (HR up to 2.24) (22). The concept of quantifying organ surface nodularity has already been translated from CT to ultrasound in hepatology for the assessment of liver fibrosis (23, 24), providing a rationale for a similar translational approach in renal imaging.

Despite these advances, prior RSN studies have relied exclusively on contrast-enhanced CT. An ultrasound-based RSN assessment could provide morphological information regarding renal surface architecture without the risks of radiation exposure and contrast material administration. As an initial step toward clinical translation, this proof-of-concept study adopted a construct validity approach—demonstrating expected associations with established clinical correlates—rather than direct cross-modality comparison against CT, which would be warranted as a subsequent translational step. This study aimed to (a) develop a standardized ultrasound-based RSN grading system, (b) evaluate its prevalence, distribution, and reproducibility in a hypertensive health screening population, (c) identify factors independently associated with ultrasound-detected RSN, and (d) investigate the association between RSN grade and renal damage in this population.

## Materials and methods

### Study design and participants

This retrospective cross-sectional study was conducted at the Taihu Lake Cadre Sanatorium of Jiangsu Province. The study protocol was approved by its institutional review board (approval number SGLL-26009), and the requirement for individual informed consent was waived owing to the retrospective design. All procedures complied with the Declaration of Helsinki.

Hypertensive adults who underwent abdominal ultrasound examination during routine health screening between October 2025 and March 2026 were assessed for eligibility (**Figure 1**). Inclusion criteria were (a) diagnosis of arterial hypertension, (b) age 18–80 years, (c) completion of renal ultrasound examination with stored coronal-plane images, and (d) availability of concurrent laboratory data (obtained within 7 days of ultrasound). Exclusion criteria were history of urinary tract infection, obstruction, or renal tuberculosis; congenital renal anomalies or hereditary nephropathies; renal cysts or masses exceeding 1 cm; prior renal surgery; visually apparent bilateral renal size asymmetry; history of renal infarction; active or prior malignancy; and history of myocardial infarction or atrial fibrillation. Myocardial infarction and atrial fibrillation were exclusion criteria because these conditions may cause focal cortical scarring or independently affect renal hemodynamics through cardiorenal mechanisms. Detailed exclusion numbers are provided in **Figure 1**.

**Figure 1:**
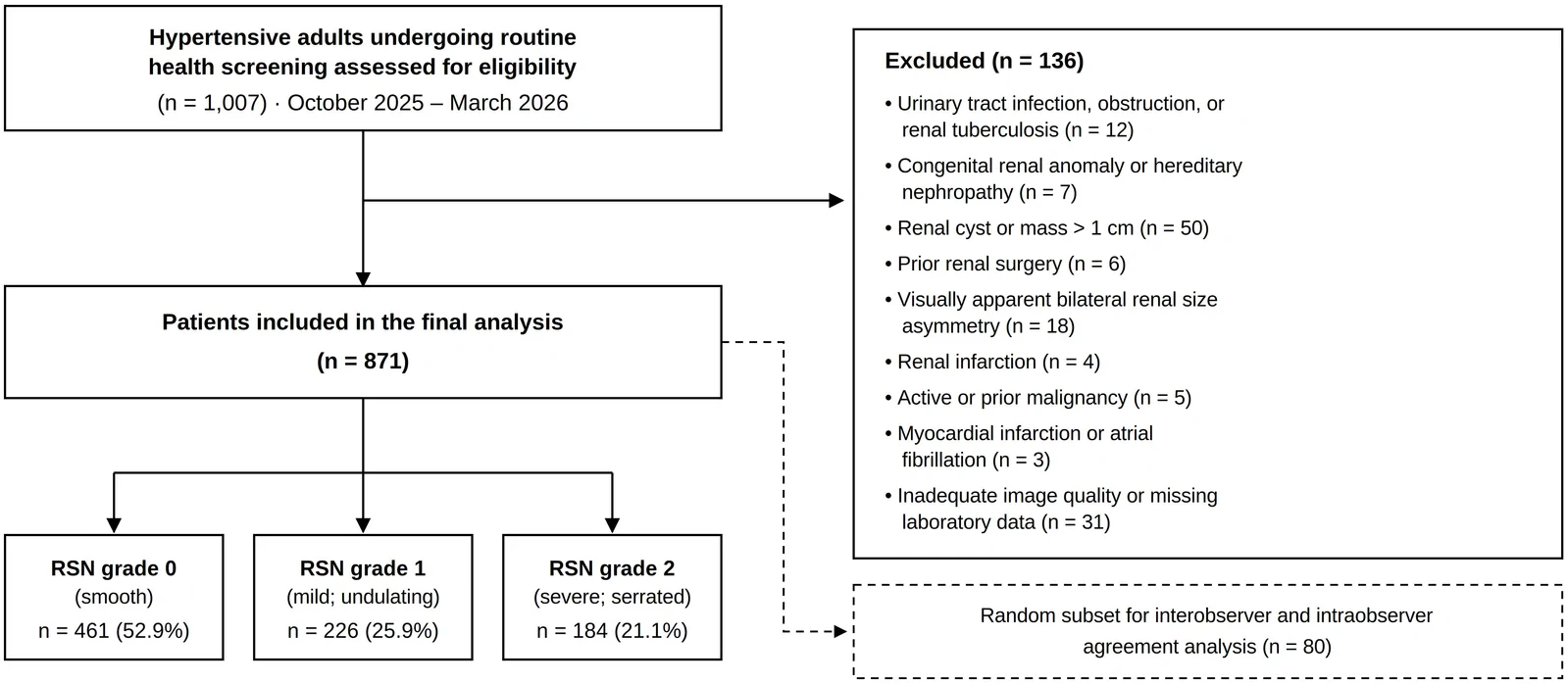
Patient selection flowchart. A total of 1007 hypertensive adults undergoing routine health screening were initially assessed for eligibility. After application of the exclusion criteria, 871 patients were included in the final analysis.

### Clinical data collection

The following variables were collected from medical records (**Table 1**): demographics; blood pressure and hypertension grade according to the 2020 International Society of Hypertension guidelines (25); hypertension duration (categorized as < 10 years, 10–20 years, or > 20 years); diabetes mellitus; and laboratory markers, including serum creatinine, eGFR (modified MDRD equation for the Chinese population), blood urea nitrogen (BUN), uric acid, urinary protein, triglycerides, total cholesterol, HDL-C, and LDL-C.

**Table 1:** Clinical characteristics of hypertensive patients stratified by RSN grade.

Variable	Overall (N = 871)	Grade 0 (n = 461)	Grade 1 (n = 226)	Grade 2 (n = 184)	Statistic	P value
Age (years)	58.0 (51.0, 69.0)	57.0 (49.0, 64.0)	59.0 (52.0, 69.0)	64.0 (55.0, 76.0)	42.62	<.001
Sex (male), n (%)	725 (83.2)	368 (79.8)	187 (82.7)	170 (92.4)	14.93	<.001
Hypertension grade, n (%)					195.00	<.001
— Grade 1	155 (17.8)	133 (28.9)	21 (9.3)	1 (0.5)		
— Grade 2	353 (40.5)	212 (46.0)	106 (46.9)	35 (19.0)		
— Grade 3	363 (41.7)	116 (25.2)	99 (43.8)	148 (80.4)		
Hypertension duration, n (%)					115.42	<.001
— < 10 years	554 (63.6)	357 (77.4)	133 (58.8)	64 (34.8)		
— 10–20 years	248 (28.5)	80 (17.4)	81 (35.8)	87 (47.3)		
— > 20 years	69 (7.9)	24 (5.2)	12 (5.3)	33 (17.9)		
Diabetes, n (%)	190 (21.8)	93 (20.2)	53 (23.5)	44 (23.9)	1.56	.459
Cortical echo intensity, n (%)					196.78	<.001
— Grade 1 (lower than liver/spleen)	362 (41.6)	264 (57.3)	78 (34.5)	20 (10.9)		
— Grade 2 (equal to liver/spleen)	334 (38.3)	145 (31.5)	118 (52.2)	71 (38.6)		
— Grade 3 (higher than liver/spleen)	175 (20.1)	52 (11.3)	30 (13.3)	93 (50.5)		
eGFR (mL/min/1.73 m²)	94.9 (83.8, 102.5)	98.9 (90.4, 106.0)	92.7 (83.9, 99.6)	79.9 (63.6, 89.9)	171.42	<.001
eGFR < 60 mL/min/1.73 m², n (%)	51 (5.9)	3 (0.7)	6 (2.7)	42 (22.8)	122.99	<.001
BUN (mmol/L)	5.8 (5.0, 6.9)	5.5 (4.8, 6.5)	5.9 (5.0, 6.7)	6.7 (5.5, 7.9)	58.10	<.001
Uric acid (μmol/L)	370.0 (319.5, 434.0)	365.0 (312.0, 424.0)	366.0 (323.2, 441.0)	391.0 (340.5, 455.5)	14.53	<.001
Elevated creatinine, n (%)	23 (2.6)	2 (0.4)	0 (0.0)	21 (11.4)	69.94	<.001
Proteinuria, n (%)	57 (6.5)	22 (4.8)	12 (5.3)	23 (12.5)	13.60	.001
Triglycerides (mmol/L)	1.5 (1.1, 2.2)	1.5 (1.1, 2.2)	1.5 (1.1, 2.3)	1.5 (1.1, 2.2)	1.06	.589
Total cholesterol (mmol/L)	4.6 (3.9, 5.3)	4.7 (3.9, 5.3)	4.7 (3.9, 5.2)	4.6 (3.8, 5.3)	0.32	.854
HDL-C (mmol/L)	1.2 (1.0, 1.4)	1.2 (1.0, 1.4)	1.2 (1.0, 1.4)	1.1 (1.0, 1.4)	0.03	.985
LDL-C (mmol/L)	2.9 (2.3, 3.5)	2.9 (2.3, 3.5)	2.9 (2.3, 3.4)	2.9 (2.2, 3.5)	0.46	.793
BMI (kg/m²)	25.9 (24.1, 28.2)	26.0 (24.1, 28.3)	25.8 (24.5, 28.1)	25.9 (24.1, 27.9)	0.13	.937
Renal damage, n (%)	92 (10.6)	24 (5.2)	18 (8.0)	50 (27.2)	69.36	<.001

—Continuous variables are presented as median (interquartile range) and were compared using the Kruskal–Wallis test (H statistic). Categorical variables are presented as n (%) and were compared using the χ² test (χ² statistic). Percentages for subcategories are column percentages within each RSN group. RSN = renal surface nodularity, Egfr, estimated glomerular filtration rate; BUN, blood urea nitrogen; HDL-C, high-density lipoprotein cholesterol; LDL-C, low-density lipoprotein cholesterol; BMI, body mass index; IQR, interquartile range.

### Ultrasound examination and RSN assessment

All renal ultrasound examinations were performed using a GE LOGIQ E11 system (GE HealthCare, Chicago, IL, USA) equipped with a C1–6 convex array transducer operating at 4.5 MHz. Patients were examined in the supine or lateral decubitus position. For each kidney, coronal-plane scanning through the renal hilum was performed and multiple frames were acquired, from which a single representative static frame was selected for grading using predefined criteria: a hilar section showing the maximal renal length, a complete and clearly delineated capsule, no appreciable respiratory-motion or reverberation artefact, and clear parenchyma–sinus differentiation. Subjects breathed quietly (tidal respiration, without breath-hold), and uniform light transducer pressure was applied to avoid contour deformation. Imaging parameters were standardized as follows: dynamic range 60–80 dB; gain adjusted to display the capsule clearly; a single near-field focal zone at the capsule; tissue harmonic and spatial compound imaging enabled; and depth (10–15 cm) adjusted to body habitus.

RSN was graded retrospectively from the stored standardized coronal-plane image by a sonographer with more than 10 years of experience who was blinded to laboratory results. Grading followed a three-point scale adapted from the previously validated CT-based system (19, 21) (**Figure 2**): grade 0 (smooth), smooth renal capsule with intact continuity; grade 1 (mild, undulating), undulating or wave-like contour, or focal indentations with a depth less than one-third of the cortical thickness; and grade 2 (severe, serrated), serrated or nodular contour with an indentation depth greater than one-third of the cortical thickness, or with accompanying cortical thinning. The specific feature qualifying a kidney for grade 2 (contour/indentation versus cortical thinning) was not recorded separately. Cortical echo intensity was classified as grade 1 (lower than liver or spleen), grade 2 (equal to liver or spleen), or grade 3 (higher than liver or spleen) (10, 11).

**Figure 2:**
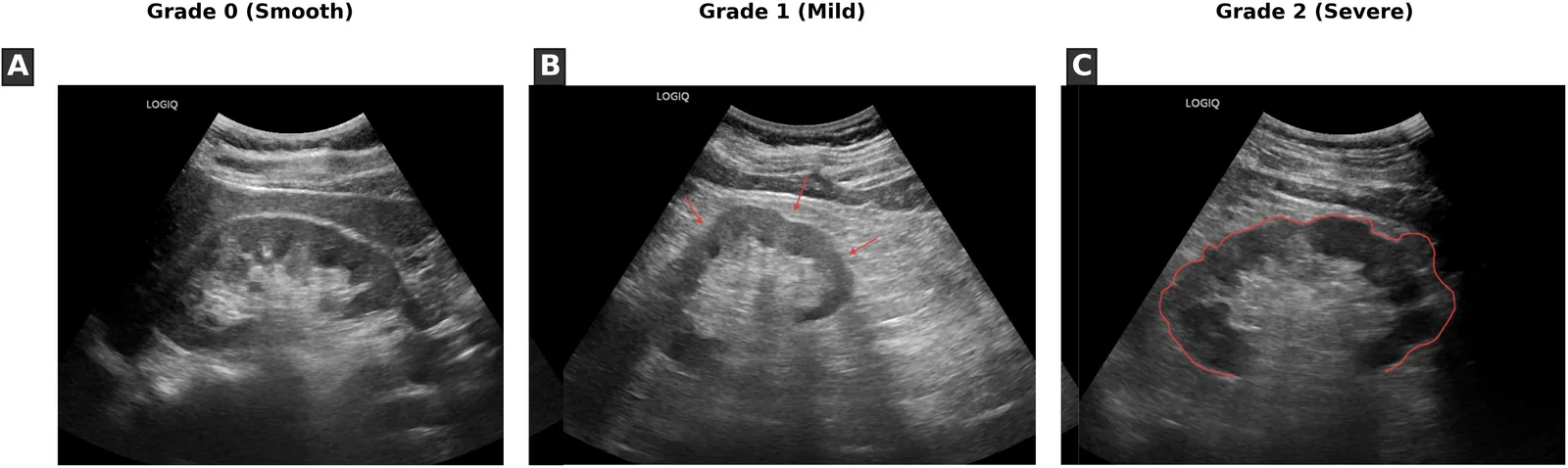
Representative ultrasound images of the three-point renal surface nodularity (RSN) grading system. **(A)** Grade 0: smooth renal capsule with intact continuity. **(B)** Grade 1 (mild): undulating contour with focal indentations (arrows). **(C)** Grade 2 (severe): serrated, nodular contour with nodules of varying size (red line).

For analysis, the maximum RSN grade of the two kidneys was used as the patient-level RSN score. This corresponds to the worse-affected kidney and was chosen *a priori* because hypertensive nephrosclerosis may be asymmetric; given the high bilateral concordance (intraobserver κ = 0.888), the choice had limited influence, and a sensitivity analysis using the mean bilateral grade was consistent (model 4 OR, 1.76 per grade; 95% CI: 1.10–2.79; P = .017).

### Interobserver and intraobserver agreement

Interobserver and intraobserver agreement were assessed in a random subset of 80 patients using stored standardized coronal-plane images from the initial examination. A second sonographer independently evaluated the images while blinded to the results of Reader 1. Reader 1 re-evaluated the same subset after an interval of at least 4 weeks. Agreement was quantified using the weighted (quadratic) Cohen κ coefficient and interpreted according to established benchmarks (26).Because both readers assessed identical stored images, these kappa values reflect the reproducibility of image interpretation rather than of image acquisition.

### Outcome definitions

Renal damage was defined as the presence of at least one of the following: (a) eGFR < 60 mL/min/1.73 m² or (b) positive urinary protein (≥ 1+), based on KDIGO 2024 criteria (5).

### Statistical analysis

Continuous variables were summarized as medians with interquartile ranges and categorical variables as frequencies and percentages. Comparisons across RSN groups used the Kruskal–Wallis test for continuous variables and the χ² test for categorical variables, and trends across ordered RSN grades were tested with the Jonckheere–Terpstra test (eGFR) and the Cochran–Armitage test (renal-damage prevalence). Spearman rank correlation coefficients were calculated to quantify associations between RSN grade and the clinical variables of interest.

The analysis comprised two components. First, binary logistic regression (grade ≥ 1 vs grade 0) was performed to identify factors associated with RSN; variables with P <.20 at univariable analysis entered the multivariable model. Multicollinearity was evaluated with the variance inflation factor (VIF > 5 indicating collinearity), and model discrimination was summarized by the AUC with a DeLong 95% CI, with calibration and optimism-corrected discrimination obtained from 1000-sample bootstrap internal validation. Second, sequential logistic regression models examined the association between RSN grade and renal damage: model 1 (unadjusted); model 2 (adjusted for age, sex, hypertension grade, and diabetes); model 3 (further adjusted for hypertension duration and cortical echo intensity); and model 4 (model 3 further adjusted for eGFR as a continuous variable). The incremental value of RSN beyond the model 4 covariates was assessed by the change in AUC (DeLong test) and the integrated discrimination improvement, for both the composite and the non-eGFR endpoints. Sensitivity analyses decomposed the composite endpoint into its individual components (**Supplementary Table S1**). In an additional sensitivity analysis, the eGFR threshold used to define renal damage was varied from 55 to 65 mL/min/1.73 m² (**Supplementary Table S2**). Two-sided P <.05 was considered statistically significant.

## Results

### Study population

A total of 871 hypertensive patients were included in the final analysis (**Figure 1**). The median age was 58.0 years (IQR, 51.0–69.0), and 725 of 871 patients (83.2%) were men. Hypertension grades were distributed as follows: grade 1, 155 patients (17.8%); grade 2, 353 (40.5%); and grade 3, 363 (41.7%). Diabetes mellitus was present in 190 patients (21.8%) (**Table 1**).

### Prevalence and bilateral concordance of ultrasound-detected RSN

RSN was detected in 410 of 871 patients (47.1%). By individual kidney, RSN was present in the left kidney in 401 patients (46.0%) and in the right kidney in 402 (46.2%). Using the maximum grade of the two kidneys, 461 patients (52.9%) were classified as grade 0, 226 (25.9%) as grade 1, and 184 (21.1%) as grade 2. Concordance between left and right kidney RSN grades was high, with agreement in 768 of 871 cases (88.2%) and a weighted κ of 0.888, indicating that RSN typically manifests bilaterally.

### Interobserver and intraobserver agreement

The reproducibility of ultrasound-based RSN grading was evaluated in a random subset of 80 patients (**Table 2**). For the patient-level RSN score, interobserver agreement yielded a weighted κ of 0.798 (95% CI: 0.693–0.887), with percentage agreement of 80.0% (64 of 80), indicating substantial agreement. Intraobserver agreement showed a weighted κ of 0.888 (95% CI: 0.808–0.952), with percentage agreement of 87.5% (70 of 80), indicating almost perfect agreement. For individual kidney assessment, both interobserver and intraobserver κ values exceeded 0.83. Cortical echo intensity also demonstrated substantial-to-almost-perfect reproducibility (interobserver κ = 0.801; intraobserver κ = 0.850).

**Table 2:** Interobserver and intraobserver agreement of ultrasound-based RSN grading and cortical echo intensity (n = 80).

Agreement type	Assessment level	Weighted κ	95% CI	Percentage agreement
Interobserver	RSN: patient level (maximum)	0.798	0.693–0.887	80.0% (64/80)
Interobserver	RSN: left kidney	0.865	0.768–0.936	86.2% (69/80)
Interobserver	RSN: right kidney	0.838	0.741–0.912	83.8% (67/80)
Interobserver	Cortical echo intensity	0.801	0.693–0.885	77.5% (62/80)
Intraobserver	RSN: patient level (maximum)	0.888	0.808–0.952	87.5% (70/80)
Intraobserver	RSN: left kidney	0.882	0.802–0.945	87.5% (70/80)
Intraobserver	RSN: right kidney	0.875	0.789–0.942	86.2% (69/80)
Intraobserver	Cortical echo intensity	0.850	0.759–0.917	82.5% (66/80)

—Weighted κ was calculated using quadratic weights, with bootstrap 95% CIs (2000 iterations). Agreement was assessed on stored standardized coronal-plane images and therefore reflects agreement in image interpretation rather than image acquisition. Interpretation of κ: **<** 0.20, poor; 0.21–0.40, fair; 0.41–0.60, moderate; 0.61–0.80, substantial; 0.81–1.00, almost perfect agreement.

### Clinical characteristics across RSN groups

The three RSN groups differed significantly in multiple clinical parameters (**Table 1**). Patients with higher RSN grades were older (median age, 57, 59, and 64 years for grades 0, 1, and 2, respectively; P <.001), were more frequently male (79.8%, 82.7%, and 92.4%; P <.001), and had higher hypertension grades and longer hypertension duration (both P <.001) (**Figure 3**).

**Figure 3:**
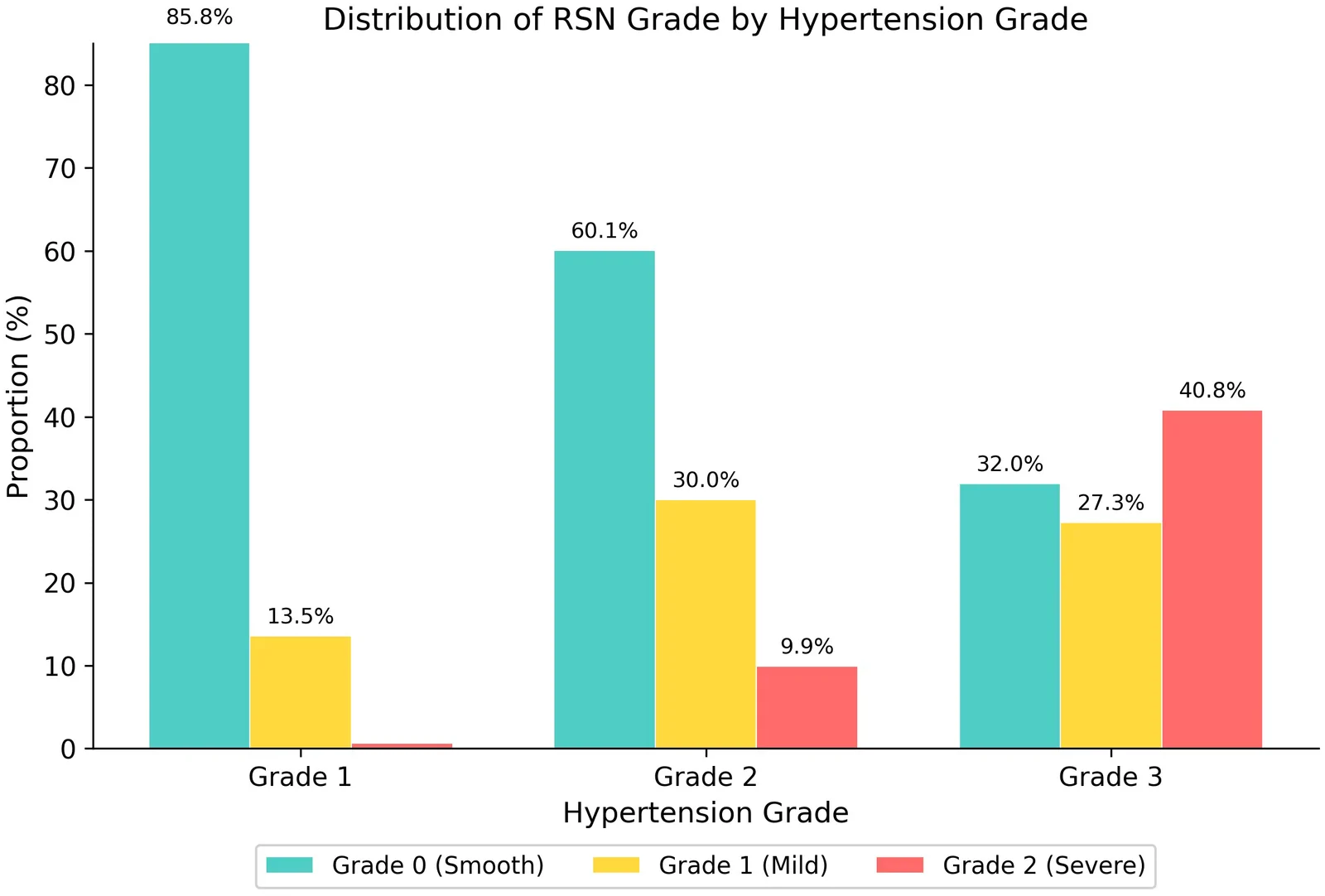
Distribution of RSN grade by hypertension grade. The proportion of grade 2 (severe) RSN increased progressively with higher hypertension grade (P <.001).

eGFR declined progressively across RSN groups (median, 98.9, 92.7, and 79.9 mL/min/1.73 m²; P <.001). The proportion of patients with eGFR < 60 mL/min/1.73 m² increased from 0.7% in grade 0 to 2.7% in grade 1 and 22.8% in grade 2 (P <.001). Proteinuria increased from 4.8% to 5.3% and 12.5% (P = .001). Cortical echo intensity grade 3 was observed in 11.3% of grade 0, 13.3% of grade 1, and 50.5% of grade 2 patients (P <.001). No significant differences were observed for lipid parameters (all P >.42) or BMI (P = .937). Spearman correlation analysis (**Figure 4**) demonstrated the strongest associations between RSN grade and hypertension grade (ρ = 0.447), eGFR (ρ = −0.434), cortical echo intensity (ρ = 0.412), and hypertension duration (ρ = 0.340; all P <.01).

**Figure 4:**
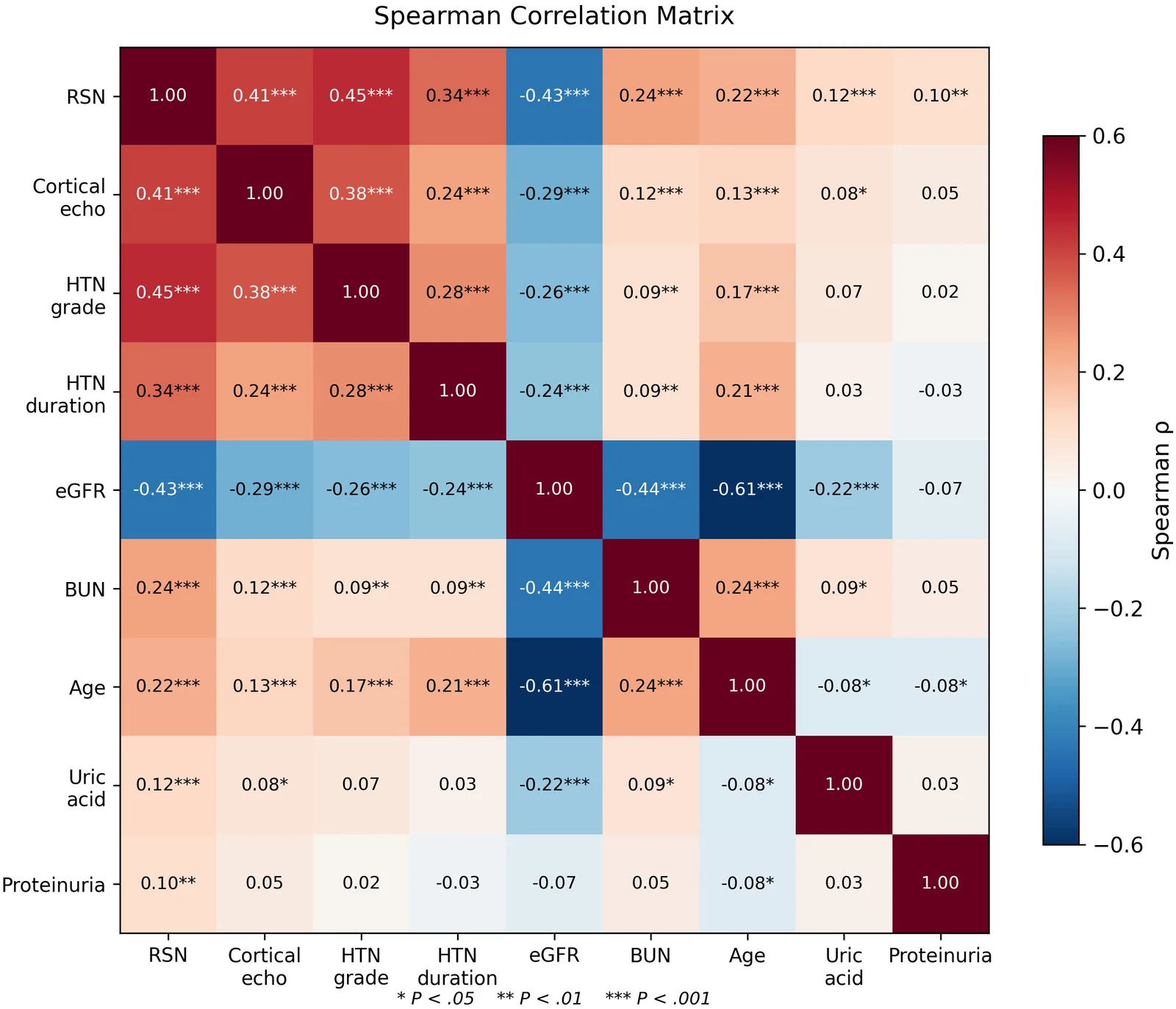
Spearman correlation matrix. RSN grade showed the strongest correlations with hypertension grade (ρ = 0.447), eGFR (ρ = −0.434), cortical echo intensity (ρ = 0.412), and hypertension duration (ρ = 0.340). *P <.05; **P <.01; ***P <.001.

### Factors associated with RSN

On univariable logistic regression, hypertension grade, cortical echo intensity, hypertension duration, proteinuria, male sex, BUN, age, uric acid, and eGFR were significantly associated with RSN (**Table 3**). On multivariable analysis (all VIF < 2.0), four factors remained independently associated with RSN: hypertension grade (OR, 2.40; 95% CI: 1.88–3.07; P <.001), cortical echo intensity (OR, 1.63; 95% CI: 1.30–2.05; P <.001), hypertension duration (OR, 1.62; 95% CI: 1.24–2.11; P <.001), and lower eGFR (OR, 0.625 per 10 mL/min/1.73 m² increase; 95% CI: 0.53–0.74; P <.001). The model showed good discriminative performance (AUC = 0.815). On bootstrap internal validation (1000 resamples), the model was well calibrated (calibration slope 0.955) with an optimism-corrected AUC of 0.806 (apparent AUC 0.815; 95% CI: 0.787-0.842).

**Table 3:** Univariable and multivariable logistic regression for factors associated with RSN (grade ≥ 1 vs grade 0).

Variable	Univariable OR	95% CI	P value	Multivariable OR	95% CI	P value
Age (per year)	1.034	1.022–1.046	<.001	0.987	0.970–1.005	.158
Male sex	1.702	1.179–2.458	.005	0.913	0.570–1.462	.705
Hypertension grade	3.450	2.771–4.294	<.001	2.401	1.878–3.069	<.001
Hypertension duration	2.524	2.001–3.183	<.001	1.620	1.244–2.111	<.001
Diabetes	1.226	0.889–1.692	.214			
Cortical echo intensity	2.665	2.189–3.246	<.001	1.631	1.298–2.049	<.001
eGFR (per mL/min/1.73 m²)	0.946	0.936–0.956	<.001	0.954	0.939–0.970	<.001
BUN (per mmol/L)	1.321	1.208–1.446	<.001	1.058	0.956–1.170	.278
Uric acid (per μmol/L)	1.002	1.001–1.004	.004	1.000	0.998–1.002	.953
Proteinuria	1.862	1.074–3.231	.027	1.788	0.857–3.730	.121
Triglycerides	0.993	0.926–1.064	.835			
Total cholesterol	0.981	0.865–1.113	.765			
HDL-C	0.816	0.586–1.136	.228			
LDL-C	0.957	0.826–1.109	.559			
BMI (per kg/m²)	0.979	0.942–1.018	.289			

—The multivariable model included variables with P **<**.20 at univariable analysis. AUC of the multivariable model = 0.815. All VIF **<** 2.0. An OR of 0.954 per 1 mL/min/1.73 m² increase in eGFR corresponds to an OR of 0.625 (95% CI: 0.53–0.74) per 10 mL/min/1.73 m² increase. AUC, area under the receiver operating characteristic curve; CI, confidence interval; OR, odds ratio; VIF, variance inflation factor.

### Association between RSN and renal damage

Renal damage was identified in 92 of 871 patients (10.6%). Its prevalence exhibited a dose–response relationship with RSN grade: 5.2% in grade 0, 8.0% in grade 1, and 27.2% in grade 2 (P for trend <.001) (**Figure 5A**). The eGFR distribution likewise showed a progressive decline across RSN groups (**Figure 5B**). The eGFR decline across ordered grades was significant (Jonckheere-Terpstra test, P <.001).

**Figure 5:**
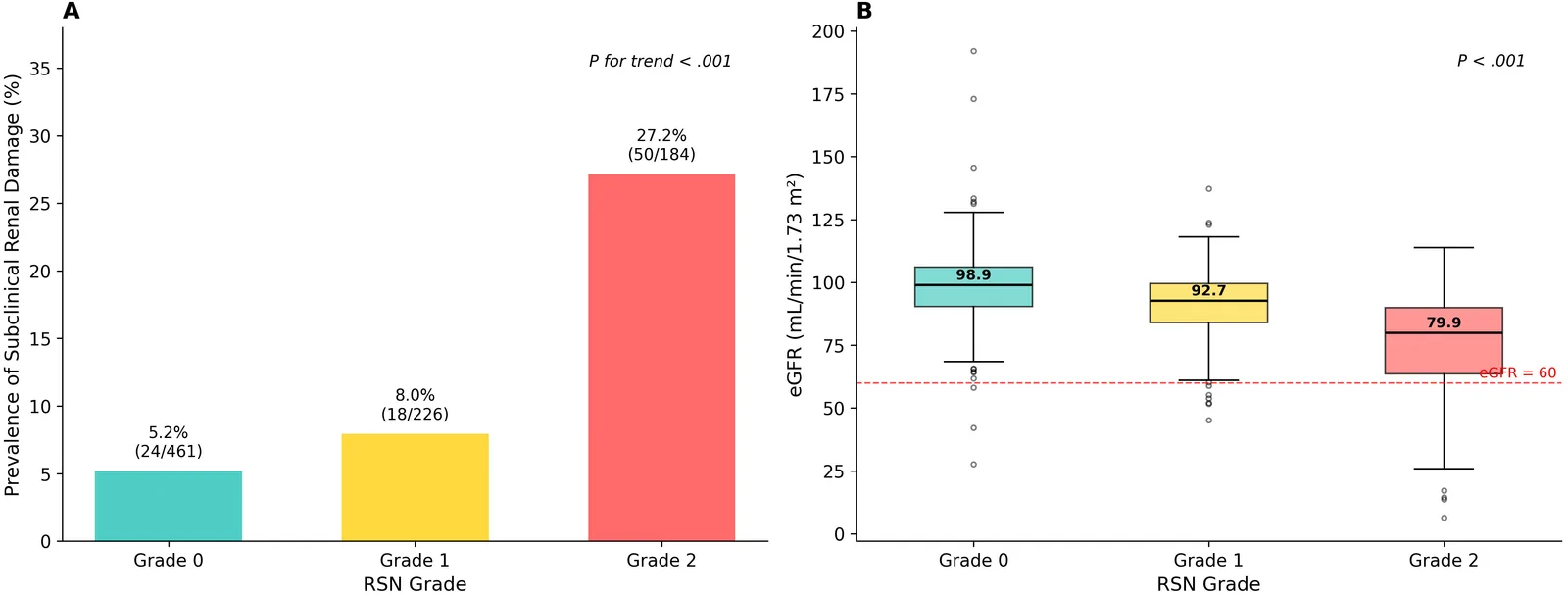
**(A)** Prevalence of renal damage by RSN grade (5.2%, 8.0%, and 27.2% for grades 0, 1, and 2, respectively; P for trend <.001). **(B)** Box plots showing the eGFR distribution by RSN grade (median, 98.9, 92.7, and 79.9 mL/min/1.73 m²; P <.001). The dashed line indicates eGFR = 60 mL/min/1.73 m².

In sequential logistic regression models (**Table 4**), grade 2 RSN was significantly associated with renal damage across all models. In the unadjusted model (model 1), grade 2 RSN was associated with an OR of 6.79 (95% CI: 4.02–11.47; P <.001). After adjustment for age, sex, hypertension grade, and diabetes (model 2), grade 2 RSN remained strongly associated (OR, 8.77; 95% CI: 4.49–17.13; P <.001); diabetes was also independently associated (OR, 2.04; P = .005), whereas hypertension grade was not (P = .075) (**Figure 6**). In model 3, grade 2 RSN remained independently associated (OR, 7.66; 95% CI: 3.71–15.84; P <.001). In model 4, which was additionally adjusted for eGFR as a continuous variable, grade 2 RSN remained associated with renal damage (OR, 2.88; 95% CI: 1.26–6.61; P = .012), although the magnitude was markedly attenuated relative to model 3. All VIF values remained below 2.0 across models.

**Table 4:** Logistic regression for the association between RSN grade and renal damage.

Model	Variable	OR	95% CI	P value
Model 1 (unadjusted)	Grade 1	1.58	0.84–2.97	.159
	Grade 2	6.79	4.02–11.47	<.001
Model 2	Grade 1	1.75	0.90–3.40	.098
	Grade 2	8.77	4.49–17.13	<.001
	Age	1.01	0.99–1.03	.188
	Male sex	0.89	0.46–1.72	.734
	Hypertension grade	0.70	0.47–1.04	.075
	Diabetes	2.04	1.25–3.33	.005
Model 3	Grade 1	1.70	0.87–3.33	.118
	Grade 2	7.66	3.71–15.84	<.001
	Age	1.01	0.99–1.03	.157
	Male sex	0.85	0.44–1.64	.624
	Hypertension grade	0.68	0.46–1.01	.055
	Diabetes	2.04	1.25–3.35	.005
	Hypertension duration	0.78	0.53–1.15	.211
	Cortical echo intensity	1.43	1.01–2.01	.042
Model 4 (model 3 + eGFR)	Grade 1	1.07	0.52–2.21	.851
	Grade 2	2.88	1.26–6.61	.012
	Age	0.96	0.93–0.98	<.001
	Male sex	0.35	0.16–0.78	.010
	Hypertension grade	0.69	0.45–1.07	.095
	Diabetes	3.30	1.83–5.94	<.001
	Hypertension duration	0.66	0.42–1.05	.082
	Cortical echo intensity	1.03	0.68–1.53	.904
	eGFR (per mL/min/1.73 m²)	0.92	0.90–0.94	<.001

—Model 1: unadjusted. Model 2: adjusted for age, sex, hypertension grade, and diabetes. Model 3: model 2 further adjusted for hypertension duration and cortical echo intensity. Model 4: model 3 further adjusted for eGFR as a continuous variable. Reference category: grade 0. All VIF **<** 2.0. Model 4 is the primary model. Because eGFR is calculated from age and sex, the age and sex coefficients in model 4 are adjustment terms only and should not be interpreted as independent effects.

**Figure 6:**
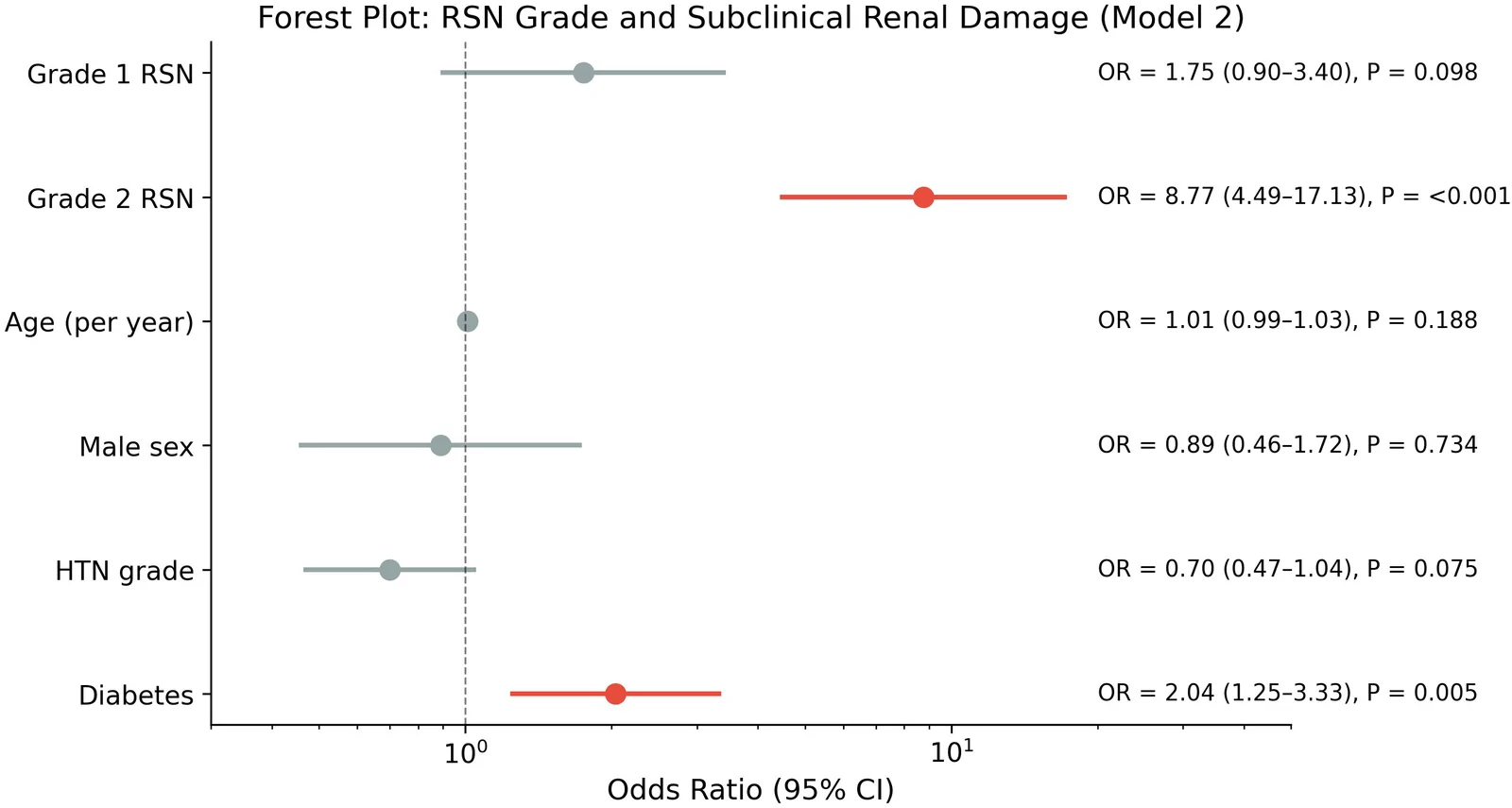
Forest plot of the association between RSN grade and renal damage (model 2). Grade 2 RSN was independently associated with renal damage (OR, 8.77; 95% CI: 4.49–17.13; P <.001).

In sensitivity analyses (**Supplementary Table S1**), grade 2 RSN was associated with eGFR < 60 mL/min/1.73 m² (adjusted OR, 41.30; 95% CI: 10.50–162.49; P <.001); the very wide confidence interval reflects the small number of events among grade 0 patients (n = 3), and the magnitude of this estimate should therefore be interpreted with caution. Grade 2 RSN was also associated with proteinuria (adjusted OR, 4.82; 95% CI: 2.20–10.55; P <.001). When eGFR < 60 mL/min/1.73 m² was excluded from the composite endpoint, grade 2 RSN remained significantly associated with the remaining components before eGFR adjustment (OR, 5.61; 95% CI: 2.71–11.63; P <.001); after additional adjustment for eGFR as a continuous variable, however, this association was no longer significant (OR, 1.55; 95% CI: 0.67–3.57; P = .30). Grade 1 RSN was not significantly associated with renal damage in any model. Varying the eGFR threshold used to define renal damage from 55 to 65 mL/min/1.73 m² yielded grade 2 odds ratios of 2.16 to 2.88, attenuating to borderline significance at the 65 mL/min/1.73 m² threshold (**Supplementary Table S2**).

### RSN in patients without threshold-defined renal damage

Among the 779 patients who did not meet the definition of renal damage (eGFR ≥ 60 mL/min/1.73 m² and negative urinary protein), RSN was nevertheless frequently observed: 437 patients (56.1%) were grade 0, 208 (26.7%) were grade 1, and 134 (17.2%) were grade 2. Conversely, 134 of the 184 patients with grade 2 RSN (72.8%) did not meet the threshold definition of renal damage. Within this subgroup, eGFR declined progressively across RSN grades (median, 98.8, 93.1, and 87.4 mL/min/1.73 m² for grades 0, 1, and 2, respectively; H = 99.00, P <.001; Spearman ρ = −0.353, P <.001), as did BUN (median, 5.6, 5.9, and 6.1 mmol/L; H = 21.76, P <.001) (**Supplementary Table S3**).

## Discussion

To our knowledge, this is the first study to systematically evaluate ultrasound-detected renal surface nodularity in a hypertensive health screening population. The principal findings are that ultrasound can detect and grade RSN with substantial-to-almost-perfect reproducibility; that RSN is independently associated with hypertension severity and decreased eGFR, and that grade 2 RSN is associated with renal damage, although this association overlaps substantially with the information conveyed by eGFR. Because participants were enrolled through routine health screening—rather than from hypertension or nephrology clinics—and patients with myocardial infarction or atrial fibrillation were excluded, the cohort likely represents a relatively low-risk population. Higher RSN grades and renal damage may thus be more frequent in clinic-based settings, where these associations warrant confirmation. In addition, because RSN was graded from a single standardized coronal-plane image, nodularity outside the imaged plane may go undetected; the grading is therefore more likely to under-detect than to over-detect RSN, and volumetric or multi-plane acquisition would improve surface sampling.

Several lines of evidence support the construct validity of ultrasound-detected RSN. RSN grade correlated most strongly with hypertension grade (ρ = 0.447), decreased eGFR (ρ = −0.434), and hypertension duration (ρ = 0.340), mirroring the clinical correlates previously reported for CT-based RSN (18–22) and consistent with the arteriolar–ischemic mechanism by which nephron loss and glomerulosclerosis produce macroscopic surface irregularity (3, 4, 14). The independent association with hypertension duration further suggests that RSN reflects the cumulative Hemodynamic burden that a single blood pressure measurement cannot capture (27). Of note, when both RSN and hypertension grade were included in the same model, hypertension grade lost its independent significance (P = .075), indicating substantial shared variance: RSN captures structural consequences of chronic hypertension that overlap with, rather than surpass, the information conveyed by blood pressure severity. The high bilateral concordance (κ = 0.888; agreement, 88.2%) accords with the diffuse and symmetric nature of hypertensive arteriolar disease and provides direct empirical support for the assumption—previously stated but untested—that RSN is comparable between the two kidneys of a given patient (20).

The reproducibility of the grading system is essential for clinical adoption. The interobserver κ of 0.798 and intraobserver κ of 0.888 are of similar magnitude to the intraclass correlation coefficients reported for CT-based quantitative RSN metrics (ICC, 0.858–0.982) in a prior study (22), although κ and ICC are not directly comparable. Agreement was somewhat higher for individual kidney assessment (κ > 0.83) than for the patient-level score.

A central finding of this study is the dose–response relationship between RSN grade and renal damage, with prevalence increasing more than fivefold from grade 0 to grade 2 (**Figure 5A**) and grade 2 RSN remaining strongly associated with renal damage in models 2 and 3. The rise in the grade 2 odds ratio from the unadjusted to the adjusted model, together with the loss of independent significance of hypertension grade, is characteristic of negative confounding (suppression): RSN grade and hypertension grade are moderately correlated (ρ = 0.447) and share outcome-related variance, so conditioning on both allows RSN to capture the renal-damage-predictive component while the residual effect of hypertension grade is attenuated. Variance inflation factors below 2.0 indicate partial variance-sharing rather than severe multicollinearity, and the precise magnitude of the grade 2 estimate should therefore be interpreted with caution. Coexisting diabetes conferred additional risk (OR, 2.04), consistent with known synergistic mechanisms of renal injury (28, 29). Grade 1 RSN showed only a nonsignificant trend toward increased renal damage, which may indicate early surface remodeling not yet detectable by creatinine-based eGFR or dipstick proteinuria.

To address potential circularity between RSN and the eGFR component of the composite endpoint, model 4 additionally adjusted for eGFR as a continuous variable. The association of grade 2 RSN persisted but was markedly attenuated (OR, 2.88; P = .012), and when the same adjustment was applied to the non-eGFR components of the endpoint, the association was no longer significant (OR, 1.55; 95% CI: 0.67–3.57; P = .30). Consistent with this, adding RSN grade to a model already containing eGFR and the model 4 covariates produced only a marginal, non-significant gain in discrimination (ΔAUC 0.009; DeLong P = .20), and the association was further sensitive to the eGFR cut-off, weakening to borderline significance at 65 mL/min/1.73 m² (OR, 2.16; P = .059; **Supplementary Table S2**). These sensitivity analyses indicate that the association between RSN and renal damage overlaps substantially with the information conveyed by eGFR. We therefore present RSN not as a marker independent of eGFR, but as a morphological correlate of hypertensive renal damage that is directly visible on routine ultrasound. The apparently protective coefficients for age and male sex in model 4 do not represent genuine protective effects. Because MDRD-estimated eGFR is calculated from age and sex, entering eGFR alongside these variables forces them to be interpreted at a fixed eGFR, which can reverse their apparent direction; they are adjustment terms only and should not be read as independent effects.

Notably, RSN was not confined to patients with established functional impairment. Among the 779 patients whose eGFR and urinalysis remained within normal limits, 134 (17.2%) already exhibited grade 2 RSN, and eGFR declined progressively across RSN grades within this subgroup (P <.001). This pattern is consistent with the hypothesis that renal surface remodeling accompanies subclinical reductions in renal function before conventional diagnostic thresholds are crossed, supporting its potential as a morphological indicator appreciable during routine screening ultrasound. Because the present design is cross-sectional, however, temporal precedence cannot be established and must be tested in longitudinal cohorts.

Cortical echo intensity was independently associated with both RSN (OR, 1.63) and renal damage (OR, 1.43), reflecting related yet distinct aspects of hypertensive renal pathology: echogenicity is thought to reflect parenchymal fibrosis, whereas RSN reflects surface morphological change. Because these two sonographic features frequently coexisted in the present cohort, we explored whether their combined assessment adds information beyond either alone. Adding RSN grade to cortical echo intensity improved discrimination for renal damage (AUC, 0.646 to 0.711; ΔAUC 0.066; DeLong P <.001), indicating that the two features carry partly complementary information.

This study has several limitations. First, the cross-sectional design precludes causal inference. Second, multicenter validation across ultrasound platforms and with additional readers is warranted; moreover, because both readers assessed the same stored images, only interpretive—not acquisition-level—reproducibility was evaluated. Third, the reader was blinded to laboratory results but not to the overall parenchymal appearance of the kidney, which may have influenced RSN grading. Fourth, renal damage was defined using a modified MDRD equation rather than the CKD-EPI 2021 equation and using dipstick proteinuria; creatinine-based eGFR is less accurate near normal GFR, and cystatin C and quantitative albuminuria—neither available in this dataset—would permit more precise ascertainment. Fifth, several covariates (e.g., smoking, antihypertensive and lipid-lowering therapy) were not recorded, so residual confounding cannot be excluded, and the absence of a normotensive control group limits inferences about the specificity of RSN to hypertension. Cross-modality validation against CT-based RSN or histopathology was not performed and remains the necessary next translational step.

## Conclusion

This cross-sectional study of hypertensive adults undergoing routine health screening shows that ultrasound can grade renal surface nodularity and that RSN grade shows a graded association with renal damage. Adjustment for eGFR markedly attenuated this association, indicating that RSN is a morphological correlate of hypertensive renal damage rather than an eGFR-independent marker. Its value lies elsewhere: RSN renders nephrosclerosis remodeling directly visible on routine ultrasound, including in patients whose functional indices remain within normal limits, where structural change would otherwise go unrecognized.

## Data Availability

The raw data supporting the conclusions of this article will be made available by the authors, without undue reservation.
