# Supplementary material for: Ultrasound-detected renal surface nodularity and renal damage in hypertensive adults undergoing routine health screening: a cross-sectional study
Source: Front Endocrinol (Lausanne). 2026 Oct 19;17:1934293. doi: 10.3389/fendo.2026.1934293 (PMC13724613; doi:10.3389/fendo.2026.1934293)
Supplement: Supplementary file 1 [file Table1.docx]

**Supplementary Table S1.** *Association between RSN grade and individual components of renal damage (model 2 covariates)*

| Endpoint | Events | Grade 1 OR | 95% CI | P value | Grade 2 OR | 95% CI | P value |
| --- | --- | --- | --- | --- | --- | --- | --- |
| eGFR < 60 mL/min/1.73 m² only | 51 | 4.03 | 0.95–17.19 | .059 | 41.30 | 10.50–162.49 | < .001 |
| Proteinuria only | 57 | 1.35 | 0.63–2.90 | .438 | 4.82 | 2.20–10.55 | < .001 |
| Proteinuria or elevated creatinine (excluding eGFR < 60) | 66 | 1.22 | 0.58–2.58 | .606 | 5.61 | 2.71–11.63 | < .001 |
| Proteinuria or elevated creatinine, with eGFR adjustment | 66 | 0.72 | 0.32–1.58 | .408 | 1.55 | 0.67–3.57 | .301 |

*Note.—All models were adjusted for age, sex, hypertension grade, and diabetes (model 2 covariates). The last row was additionally adjusted for eGFR as a continuous variable. Reference category: grade 0. For the* *“****eGFR*** *<* ***60 only****”* *endpoint, only 3 events occurred among grade 0 patients; the resulting odds ratio is imprecise, and its magnitude should be interpreted with caution.*

**Supplementary Table S2.** *Sensitivity analysis: association between RSN grade and renal damage across eGFR thresholds (model 4 covariates)*

| Endpoint definition | Events | Grade 1 OR | 95% CI | P value | Grade 2 OR | 95% CI | P value |
| --- | --- | --- | --- | --- | --- | --- | --- |
| eGFR < 55 mL/min/1.73 m² or proteinuria | 81 | 1.04 | 0.50–2.18 | .920 | 2.64 | 1.13–6.14 | .025 |
| eGFR < 60 mL/min/1.73 m² or proteinuria (primary) | 92 | 1.07 | 0.52–2.21 | .851 | 2.88 | 1.26–6.61 | .012 |
| eGFR < 65 mL/min/1.73 m² or proteinuria | 109 | 1.20 | 0.61–2.36 | .605 | 2.16 | 0.97–4.78 | .059 |

*Note.—All models were adjusted for age, sex, hypertension grade, diabetes, hypertension duration, cortical echo intensity, and eGFR as a continuous variable (model 4 covariates). Reference category: grade 0.*

**Supplementary Table S3.** *Renal function indices by RSN grade among patients without threshold-defined renal damage (n = 779)*

| Variable | Grade 0 (n = 437) | Grade 1 (n = 208) | Grade 2 (n = 134) | Statistic | P value |
| --- | --- | --- | --- | --- | --- |
| Proportion of subgroup, n (%) | 437 (56.1) | 208 (26.7) | 134 (17.2) | — | — |
| eGFR (mL/min/1.73 m²) | 98.8 (90.4, 105.7) | 93.1 (84.4, 99.9) | 87.4 (75.5, 95.6) | 99.00 | < .001 |
| BUN (mmol/L) | 5.6 (4.8, 6.5) | 5.9 (5.0, 6.6) | 6.1 (5.4, 7.2) | 21.76 | < .001 |

*Note.—The subgroup comprises the 779 patients with eGFR ≥ 60 mL/min/1.73 m² and negative urinary protein (ie, those not meeting the study definition of renal damage). Continuous variables are presented as median (interquartile range) and were compared using the Kruskal–Wallis test (H statistic). Spearman correlation between RSN grade and eGFR within this subgroup: ρ = −0.353 (P* ***<*** *.001). Of the 184 patients with grade 2 RSN in the full cohort, 134 (72.8%) fall within this subgroup.*
